# Foreign ownership, subjective willing, and internal control level

**DOI:** 10.1016/j.heliyon.2024.e32970

**Published:** 2024-06-13

**Authors:** Yanxin Yi, Qin Yao, Wunhong Su

**Affiliations:** a School of Accounting, Hangzhou Dianzi University, 1158, No.2 Street, Xiasha Higher Education Zone, Hangzhou, Zhejiang, 310018, China; b International College, Krirk University, No. 3 Soi Ramindra 1, Khwaeng Anusawari, Khet Bang Khen, Krung Thep Maha Nakhon, Bangkok, 10220, Thailand

**Keywords:** Foreign ownership, Internal control level, Internal control willingness, Sustainable development, Machine learning

## Abstract

Foreign shareholders are essential in the capital market. The study on A-share listed firms from 2012 to 2021 examines the impact of foreign ownership on internal control and its transmission effect. Using text analysis and machine learning methods, we construct a variable named “internal control willingness” to explore the impact of subjective willingness. The findings indicate that foreign shareholding significantly enhances internal control quality, with a more pronounced effect observed in samples demonstrating a more positive internal control willingness. Moreover, foreign shareholders contribute to the invested firm's sustainable development by enhancing internal control quality. Further research demonstrates that the positive impact of foreign shareholding is more significant in firms with legal foreign shareholders, highly competitive industries, and sound legal environments. These findings can aid host country firms in more efficiently leveraging foreign resources and provide empirical evidence for opening up China's capital market and formulating foreign investment regulations.

## 1 Introduction

Today, no country can afford to isolate itself from development due to the irreversible trend of economic globalization. From China's first legislation, known as the “Law of the People's Republic of China on Chinese-Foreign Equity Joint Ventures” in 1979, which initially permitted only the establishment of Sino-foreign joint ventures with limited investment scope, to the formal implementation of the “People's Republic of China Foreign Investment Law” in 2020, China has undergone a historic transformation from a semi-closed economy to a fully open one. Moreover, China has consistently dedicated itself to building a more advanced framework of openness to the world, creating a favorable business environment for all firms, including multinational firms [1].

With reduced investment barriers, cross-border capital flows surge, particularly from developed nations toward emerging markets [2]. The presence of foreign investors has brought mature technological expertise, advanced management capabilities, and extensive governance know-how [3]. Motivated to safeguard their interests, investors actively participate in the host country firm's governance, aiming to bolster external oversight and enhance the quality of board-led strategic initiatives [4,5]. However, foreign shareholders encounter information asymmetry stemming from geographical, cultural, and economic differences. Considering the substantial supervision costs, foreign investors may withdraw from corporate governance within invested firms. Hence, an unanswered question remains: can foreign ownership profoundly impact the host country's internal control and consequently contribute to sustainable development?

Internal control, integral to corporate governance, is essential for fostering sustainable development. Proficient internal control not only reduces operational risks and boosts operational efficiency [6] but also bolsters sustainable development capacities. Increasingly, stakeholders recognize the importance of sustainable development, particularly in environmental sustainability, social responsibility, and corporate governance. Firms with sustainable development capabilities are strategically positioned to achieve sustained long-term growth in a dynamic market [7,8]. Consequently, sustainable development is seen as the guiding principle for future corporate advancement. ESG (Environmental, Social, and Governance) acts as a vital indicator for assessing sustainable development, signaling a shift from profit-driven to sustainable practices.

**Fig. 1:**
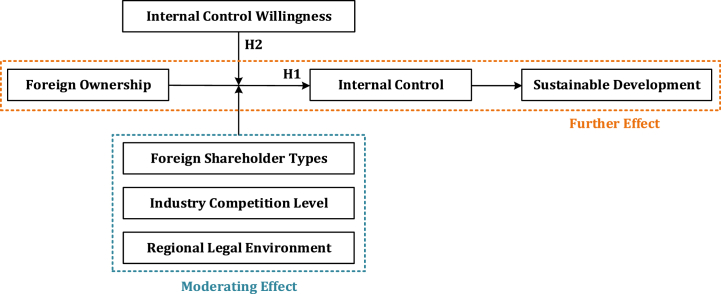
The research framework.

Therefore, this study uses available data from China's A-share market from 2012 to 2021 to investigate the impact of foreign ownership on internal control and performance concerning sustainable development, with a focus on ESG. Secondly, internal control willingness, reflecting managerial initiative in devising and implementing internal control initiatives, may moderate the relationship between foreign ownership and internal control. Furthermore, the study conducts heterogeneous analysis to explore the effects of different types of foreign shareholders, industry competitiveness, and regional legal environment. Fig. 1 illustrates the research framework.

This study makes three main research contributions. Firstly, it presents a practical method for measuring internal control willingness based on text analysis and machine learning. While Liu and Li (2021) [9] introduced the concept of “internal control willingness” and measured it through the KMeans algorithm, their method only considered the positive tone. Therefore, our research constructs an “emotion word bag” encompassing both positive and negative perspectives, enabling sentiment analysis of internal control self-assessment reports. Secondly, it expands research in internal control, particularly in the field of willingness. Internal control self-assessment reports are essential tools for assessing internal control willingness, but prior research is relatively limited. Thus, this paper focuses on the content characteristics to explore the influence of governance's subjective willingness. Thirdly, it offers fresh insights into the beneficial effects of foreign ownership, examining ownership proportion and shareholder types comprehensively. Unlike prior literature concentrating solely on proportional aspects, this research conducts a thorough analysis of foreign ownership's influence. Additionally, it provides empirical evidence for facilitating foreign investment channels in China and encouraging foreign investors to pursue long-term investment strategies.

The remainder of this paper is organized as follows. Section 2 reviews the literature. Section 3 postulates hypotheses. Section 4 details our research methodology. Sections 5, 6 present empirical findings and include robustness tests. Section 7 further explores the impact of foreign ownership on sustainable development and elaborates on heterogeneity tests. Section 8 offers a conclusion.

## 2 Literature review

The research on the economic impact of foreign ownership has been extensive, but the existing literature has not yet reached a consensus on the “governance effect” or “profit-seeking effect” of foreign ownership. On the one hand, foreign investors have demonstrated their capability to positively influence the sustainable development of emerging markets across various dimensions [10,11]. These impacts encompass reducing corporate excess perks [12], improving financial reporting quality [13,14], enhancing innovation capabilities [15], bolstering operational performance [16,17], and promoting social responsibility [18]. Previous studies also suggest that foreign investors effectively serve as supervisors in host country firms [19], contributing to greater information transparency [20,21] and more sustainable corporate governance mechanisms [22]. On the contrary, drawing upon the theory of comparative advantage, foreign shareholders are driven by profit-seeking motives. The profit-seeking tendencies of capital may reveal certain adverse consequences. For example, foreign direct investment exploits China's relaxed regulations, leading to the outsourcing of pollution and consequently exacerbating urban PM2.5 pollution levels [23,24], thereby affirming the “Pollution Heaven” hypothesis. According to Caixe et al. (2024) [25], foreign institutional investors might induce controlling shareholders to adopt short-term strategies detrimental to company value, aligning with the “locust foreign capital” perspective. Therefore, exploring the impact of foreign ownership on the development of Chinese firms holds significant importance for the efficient utilization of foreign investment resources.

Regarding the relationship between foreign ownership and internal control, Li et al. (2021) [5] discovered that companies with qualified overseas institutional investors generally exhibit higher internal control quality and fewer internal control deficiencies. Effective internal control has increasingly proven its crucial role in mitigating operational risks [26,27], enhancing profitability [6,28], curbing earnings management [29,30], promoting green innovation [31], and creating sustainability performance [32]. Additionally, factors influencing internal control can be categorized into internal and external aspects. Internal factors primarily refer to firm culture, governance structures, and operational conditions [[33], [34], [35]]. Weak internal control correlates with lower accrual quality, and a higher level of earnings management may undermine the effectiveness of the internal control system [36,37]. External factors encompass aspects such as digital transformation [38] and market environment [39].

Internal control willingness, a branch of internal control, has garnered limited attention in current research. Existing studies suggest that proactive internal control willingness can mitigate managerial overconfidence [9] and improve internal control quality. As for the evaluation of internal control willingness, it is important to recognize that internal control disclosures can indirectly reflect the management and resource allocation pertaining to a company's internal control [40]. Therefore, the “Internal Control Self-Assessment Report” is regarded as a vital source for measuring internal control willingness.

In summary, internal control and foreign ownership have been prominent areas of academic research with ample literature. However, much literature treats internal control as a mediating variable within the research on foreign ownership, providing indirect evidence of their relationship, with few studies delving into the transmission effects subsequently. Secondly, existing research lacks consensus on the economic consequences of foreign ownership, namely the “governance effect” and the “profit-seeking effect”. Moreover, research on internal control willingness is relatively limited. While internal control self-assessment reports are commonly used, the previous study primarily focuses on whether internal control deficiencies are disclosed. Tone, as one of the important characteristics of textual reports, directly reflects management's thoughts. To fully exploit the textual information, this paper employs text analysis methods to assess governance willingness conveyed in internal control self-assessment reports, thereby exploring its potential impact on the operational development of listed companies.

## 3 Theoretical analysis and research hypotheses

### 3.1 Foreign ownership and internal control level

With the rise of economic globalization, capital flows between countries have become increasingly active, with foreign investors seizing profitable opportunities overseas to diversify their investment portfolios [41]. The inflow of diverse resources empowers local firms to broaden their resources and acquire technological insights globally, affording them enduring competitive advantages and significant momentum for sustainable development [10,22]. This study aims to investigate the impact of foreign ownership on internal control, particularly focusing on governance capacity and motivation.

From a capacity perspective, most foreign investments stem from developed countries, which come with mature governance experience, substantial financial support, advanced technical resources, and professional supervisory abilities [15]. According to the resource-based theory, companies are comprised of diverse resources that are not easily transferable and difficult to replicate [42]. Their sustained competitive advantage arises from the unique resources and capabilities they possess. As a result, the wealth of governance experience often fosters a demonstration effect that prompts listed companies to address internal control deficiencies [43]. Moreover, the opening up of capital markets helps alleviate financing challenges [44], providing a stable funding source. Diverse governance structures also facilitate rapid learning of advanced technology [45]. For instance, software from overseas like SAP and Oracle have evolved from “information silos” to “system integration”, markedly boosting the efficiency of internal control implementation. Consequently, investors actively exploit technology spillovers to bring frontier insights for achieving high-quality internal control. Moreover, foreign shareholders possess professional supervisory abilities [46]. Freed from complex interpersonal relationships, foreign shareholders maintain a more independent stance, enabling them to supervise the operating activities [47] better.

From a motivation perspective, foreign investment is primarily driven by profitability. A sound internal control system can curb non-compliant behavior and drive overall development goals [48]. The goals of host country firms align with the foreign investment objectives, which drive both to achieve good cooperation and jointly strive to improve internal control quality. Additionally, foreign shareholders are notably characterized by being relatively unfamiliar with the local environment [49,50]. Due to differences in geographical distance, language, and economic conditions, investors face severe information asymmetry and high communication costs. Foreign investors find it more challenging to grasp the true situation of the firm and are thus more willing to supervise and safeguard their investment funds [51] strictly.

**Fig. 2:**
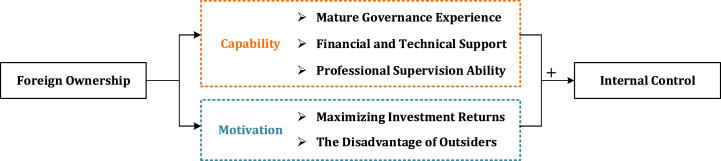
The theoretical analysis framework.

Overall, foreign investors possess both the motivation and capability to enhance internal control, taking advantage of their resources, technology, and experience to establish an effective internal control system independently. Fig. 2 displays the theoretical analysis framework.

The percentage of foreign ownership reflects the degree of influence exerted by foreign investors on invested firms and holds significant implications for their behavior [52]. In general, a lower ownership percentage implies that foreign shareholders may be more prone to short-termism [53], paying less attention to the company's sustainable development. Conversely, a higher ownership percentage indicates that foreign shareholders are more likely to demonstrate a positive impact and actively participate in the governance of the invested companies [54]. Even if the approach of “voting with hands” fails to yield the desired outcomes, considering the potential impact of foreign shareholders' withdrawal, investors can still indirectly influence the host country firms [21]. Based on the above analysis, the first hypothesis is proposed.

H1 The higher the percentage of foreign ownership, the higher the quality of internal control in host country firms.

### 3.2 The moderating effect of internal control willingness

In line with the concept of “willingness”, internal control willingness can be understood as the subjective initiative a firm exhibits in constructing and implementing its internal control activities [40]. According to modern psychology, behavior is always driven by one or more motivations. Prior research indicates that the effectiveness of internal control is influenced by the soft factors of the board of directors, supervisory board, and management, or the “human” element [55]. In other words, the subjective willingness of the governance determines the degree to which internal control objectives are achieved [56].

Firstly, internal control willingness directly impacts a company's risk perception and the establishment of preventive mechanisms [40]. Companies with a desire to improve internal control are more likely to foster prudent risk awareness. It will enable them to identify and address potential issues early on, thus preventing the accumulation of risks beyond control [57]. Secondly, this subjective initiative also encourages the governing body to initiate a “top-down” motivational effect, guiding management and even ordinary employees to voluntarily engage in efforts to improve internal control [9] and effectively prevent fraudulent activities, thereby creating a well-organized control environment and achieving stable and healthy development.

Therefore, in companies with positive internal control willingness, the board of directors is inclined to actively seek constructive suggestions from foreign shareholders regarding the development of the internal control system. Additionally, they are likely to strongly support the external oversight role of overseas investors over all levels of employees, thereby jointly striving to enhance the overall level of internal control. As a result, the second hypothesis is proposed:

H2 In firms with a positive internal control willingness, the positive impact of foreign ownership on internal control is more pronounced.

## 4 Research design

### 4.1 Data sources

Given China's update of industry classification standards in 2012, we initially utilized available data from the Chinese A-share market spanning 2012 to 2021 as a research sample. The original data underwent the following treatments: excluding firms in the financial sector, ST and *ST companies, and those with abnormal or missing data. To mitigate outlier influence on regression outcomes, we conducted winsorization on continuous variables, capping them at the 1 % critical value. These processes resulted in a final dataset of 26056 samples.

Our data sources are as follows: first, Shenzhen DIB Internal Control and Risk Management Database, accessed to obtain the “DIB China Listed Firm Internal Control Index”; second, the CSMAR Database, used for acquiring indicators related to foreign ownership, internal control assurance opinion, and control variables; third, the CNINFO website (www.cninfo.com.cn), used to acquire internal control self-assessment reports of listed companies.

### 4.2 Measurement of Internal Control Willingness

Due to the subjective nature of willingness, our study used text analysis and machine learning techniques to construct the internal control willingness variable.

#### 4.2.1 Collection of internal control self-assessment reports

Initially, we conducted a search using the keyword “Internal Control Self-Assessment Report” between 2013 and 2022.^1^ Then, we manually filtered out documents containing non-relevant terms such as “quarterly,” “semi-annual,” and “assurance opinion”, identifying annual internal control self-assessment reports meeting our criteria. Subsequently, we utilized Python 3.10 web scraping capabilities to obtain these documents, obtaining 13767 valid samples and employing the PDFMiner tool to extract the content from these documents and convert it into a manageable TXT format.

#### 4.2.2 Construction of “Positive Emotion Word Bag” and “Negative Emotion Word Bag”

Next, we utilized the “Jieba” Chinese word segmentation module to segment the report text automatically. Recognizing that certain high-frequency words like “company” and “control” may not directly relate to willingness, we referred to the “bag of words” by Liu and Li (2021) [9], along with Dalian University of Technology's Chinese Emotional Ontology and Tsinghua University's Chinese Dictionary. Subsequently, we collaborated with experts to conduct manual sentiment analysis based on linguistic conventions and potential Chinese contexts. For instance, words like “improve,” “enhance,” and “promote” indicate positive willingness, while “invalid” and “fraud” represent negative willingness. After the sentiment analysis, we extracted the top 50 words that best represented positive or negative willingness, forming the “Positive Emotion Word Bag” and “Negative Emotion Word Bag”.

#### 4.2.3 Machine learning using the K-means algorithm

**Fig. 3:**
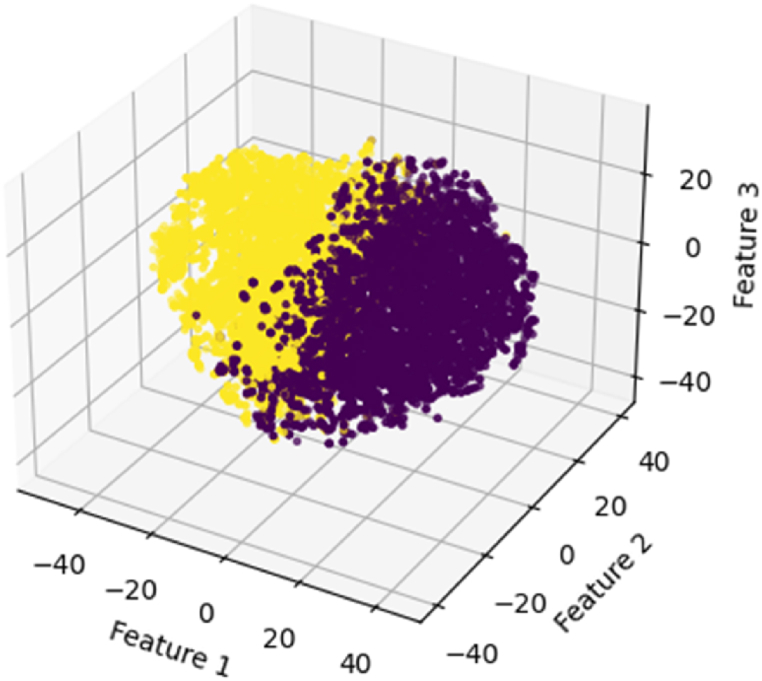
The visual clustering results.

Thirdly, we employ the K-means algorithm to build a machine-learning model. Here is how it works: First, vectorize text. Based on Python's Scikit-Learn library, we define vector dimensions based on the frequency of keywords from both the “Positive Emotion Word Bag” and the “Negative Emotion Word Bag”, converting textual data into numerical data suitable for analysis. Second, cluster. With two cluster centers representing “positive willingness” and “negative willingness,” respectively, the K-means algorithm categorizes the 13767 samples based on their internal control willingness types. Fig. 3 illustrates the visual clustering results analyzed using the K-means algorithm.^2^ Finally, a virtual variable named “internal control willingness” will be created. It is generated from the results of the clustering outcomes. Companies displaying positive willingness towards internal control are assigned a value of 1, while those predicted to have negative willingness or those with undisclosed annual internal control self-assessment reports receive a value of 0. This approach enables this study to categorize firms based on their internal control willingness, providing valuable insights for our analysis.

To validate the machine learning results, we follow the logic of the dictionary method to statistically analyze the frequency of keywords from the “Positive Emotion Word Bag” in each text, as well as the total word count. Using the ratio of the sum of keyword occurrences to the total number of words, we determine whether the self-assessment report exhibits a “positive willingness”. Subsequent testing reveals that the “predictions” derived from machine learning align closely with the outcomes obtained through the dictionary method, affirming the validity of the internal control willingness variable construction.

### 4.3 Variable definitions

Internal control level (*IC*). The “DIB China Listed Firm Internal Control Index” is widely applied to assess a firm's internal control quality [58]. To mitigate scale bias, this study takes its natural logarithm to standardize the index.

Foreign shareholding percentage (*FSHARE*). Consistent with common academic practices [12,59], we measure the extent of foreign ownership by aggregating the ownership percentages of shareholders categorized as “foreign legal entities” or “foreign natural persons” among the top ten shareholders of the company.

Internal control willingness (*POSITIVE*). Using text analysis and machine learning techniques, we create a virtual variable to quantify internal control willingness. The “Measurement of Internal Control Willingness” section details the specific measurement method.

Control Variables. Previous research has identified several critical factors influencing foreign investment decisions, including the company's size, governance structure, leverage level, and growth potential [60,61]. Therefore, our study controls for firms' operational characteristics, growth capacity, and equity structure. The selected control variables encompass firm size (*SIZE*), asset-liability ratio (*LEV*), firm age (*AGE*), return on equity (*ROE*), price-to-book ratio (*PB*), Tobin's Q (*TOBINQ*), ownership percentage of top five shareholders (*TOP5*), board size (*BOARD*), independent director ratio (*INDEP*), equity balance level (*BALANCE*), and CEO-Chairman duality (*DUAL*). Detailed measurement methods for each main variable are provided in Table 1.

**Table 1:** Variable definitions.

Variable	Measurement Method
*IC*	The natural logarithm of the “DIB China Listed Firm Internal Control Index”.
*FSHARE*	The cumulative ownership percentage of shareholders categorized as “foreign legal entities” or “foreign natural persons” among the top ten shareholders of the firm.
*POSITIVE*	This study uses text analysis and machine learning techniques to define positive internal control willingness as 1; otherwise, it is designated as 0.
*SIZE*	The natural logarithm of the firm's total assets.
*LEV*	The ratio of the firm's total liabilities to total assets.
*AGE*	The natural logarithm of the firm's years in existence plus 1.
*ROE*	The ratio of the firm's net profit to net assets.
*PB*	The ratio of a firm's per-share market price to per-share net assets.
*TOBINQ*	The ratio of year-end market capitalization, the amount of non-tradable shares as a percentage of net assets, and the liabilities to total assets.
*TOP*5	The cumulative ownership percentage of the top five shareholders of the firm.
*BOARD*	The number of the firm's board of directors.
*INDEP*	The ratio of the number of independent directors to the number of board of directors.
*BALANCE*	The ratio of the sum of the ownership percentages of the 2nd to 10th largest shareholders to the ownership percentage of the largest shareholder.
*DUAL*	When the chairman of the board and the CEO are the same person in the firm, the variable is set to 1; otherwise, it is set to 0.

### 4.4 Model construction

This study utilizes ordinary least squares regression and the Heckman two-stage method to construct Model (1), examining the impacts of foreign ownership on internal control quality. In Model (1), *IC* represents the internal control level, *FSHARE* signifies the foreign shareholding percentage, and *Control* denotes the control variables. ∑INDUSTRY and ∑YEAR are used to control for year and industry effects.

(1) $$\begin{array}{c} {IC}_{i,t}=\alpha_{0}+\alpha_{1}{FSHARE}_{i,t}+\alpha_{2}{Control}_{i,t}+\sum INDUSTRY+\sum YEAR+\varepsilon_{i,t} \end{array}$$

To investigate the moderating effect of internal control willingness, this study generates the interaction variable between foreign shareholding percentage (*FSHARE*) and internal control willingness (*POSITIVE*), denoted as (*F_POSITIVE*), which is subsequently integrated into the regression Model (2).

(2) $${IC}_{i,t}=\alpha_{0}+\alpha_{1}{F_{-}POSITIVE}_{i,t}+\alpha_{2}{FSHARE}_{i,t}+\alpha_{3}{POSITIVE}_{i,t}+\alpha_{4}{Control}_{i,t}+\sum INDUSTRY+\sum YEAR+\varepsilon_{i,t}$$

### 4.5 Descriptive statistical results

Table 2 presents the descriptive statistical results of the key variables. The internal control level (*IC*) across various firms exhibits dissimilar characteristics, ranging from 5.723 to 6.715, with a median of 6.499 and a standard deviation of 0.140. As shown in Table 2, the average value of *FSHARE* stands at 0.040, suggesting that foreign shareholders may not have substantial influence solely based on their shareholding level. However, upon analyzing the subset of foreign ownership, the average value rises to 0.125, signifying that these foreign shareholders possess the potential to exert influence on corporate governance within host countries. Additionally, the average value of internal control willingness (*POSITIVE*) is 0.207, meaning that, according to the study's definition, approximately 20.7 % of listed companies are regarded as exhibiting positive willingness. In terms of control variables, the median firm size (*SIZE*) is 22.086, with a standard deviation of 1.297. The average of the asset-liability ratio (*LEV*) is 0.422, suggesting that, on average, sample firms have an asset-liability ratio of 42.2%. Descriptive statistics for the remaining control variables are also reasonable, providing ample opportunities for comparisons and analysis in our study.

**Table 2:** Descriptive statistical outcomes of the key variables.

Variable	N	Mean	SD	Min	Median	Max
*IC*	26056	6.473	0.140	5.723	6.499	6.715
*FSHARE*	26056	0.040	0.108	0.000	0.000	0.624
*POSITIVE*	26056	0.207	0.405	0.000	0.000	1.000
*SIZE*	26056	22.273	1.297	19.920	22.086	26.298
*LEV*	26056	0.422	0.202	0.057	0.415	0.873
*AGE*	26056	3.120	0.241	1.792	3.135	4.159
*ROE*	26056	0.059	0.120	−0.634	0.068	0.310
*PB*	26056	3.520	3.034	0.555	2.618	19.240
*TOBINQ*	26056	2.090	1.387	0.853	1.642	8.952
*TOP5*	26056	0.532	0.152	0.201	0.533	0.881
*BOARD*	26056	8.514	1.683	3.000	9.000	18.000
*INDEP*	26056	0.377	0.056	0.143	0.364	0.800
*BALANCE*	26056	2.581	3.413	0.248	1.357	21.029
*DUAL*	26056	0.286	0.452	0.000	0.000	1.000

## 5 Empirical analysis

### 5.1 Foreign ownership and internal control level

We conduct the Ordinary Least Squares (OLS) method and Heckman two-stage model to examine the impact of foreign ownership on the invested firm's internal control level. Table 3 represents the main results of the main test. According to the OLS regression, after controlling for relevant factors and simultaneously accounting for industry and year effects, the coefficient of *FSHARE* is 0.020, significantly positive at the 1 % level. Moreover, in the Heckman two-stage model test, the coefficient of *IMR* in the first stage is significantly greater than 0, indicating the presence of self-selection bias in the sample. After controlling for selection bias, the results in columns (3) and (4) demonstrate that the coefficients of *FSHARE* remain prominent. The above results reveal a positive correlation between foreign ownership and internal control level. With an increasing proportion of foreign shareholding among the top ten shareholders, the involvement of foreign investors facilitates invested firms in acquiring sustainable and leading competitive advantages. It is accomplished by demonstrating technology spillover effects and governance expertise, consequently enhancing the quality of internal control. In conclusion, foreign shareholders contribute positively to the internal control level of host country companies, thus corroborating hypothesis 1.

**Table 3:** The results of foreign ownership on internal control level.

Variable	OLS		Heckman	
	（1）	（2）	（3）	（4）
*FSHARE*	0.022***	0.020***	0.027***	0.020**
	(3.180)	(2.979)	(3.215)	(2.348)
*IMR*			0.172***	0.092***
			(6.077)	(5.188)
*SIZE*	0.013***	0.019***	0.067***	0.049***
	(14.917)	(20.026)	(8.717)	(10.398)
*LEV*	−0.031***	−0.039***	−0.125***	−0.084***
	(-5.231)	(-6.266)	(-6.625)	(-6.036)
*AGE*	−0.011***	−0.019***	−0.012**	−0.006
	(-3.312)	(-5.268)	(-2.038)	(-1.110)
*ROE*	0.388***	0.373***	0.470***	0.441***
	(36.641)	(35.069)	(20.982)	(20.220)
*PB*	0.001	0.001	0.002*	0.003**
	(0.983)	(0.688)	(1.792)	(2.321)
*TOBINQ*	−0.002	−0.000	0.015***	0.009***
	(-1.414)	(-0.253)	(4.855)	(3.525)
*TOP5*	0.055***	0.053***	0.166***	0.105***
	(9.222)	(8.662)	(7.989)	(6.520)
*BOARD*	0.002***	0.000	−0.000	−0.000
	(2.851)	(0.177)	(-0.469)	(-0.083)
*INDEP*	0.042**	0.024	−0.032	−0.024
	(2.407)	(1.391)	(-1.047)	(-0.831)
*BALANCE*	0.000	−0.000	−0.003***	−0.002***
	(0.889)	(-0.687)	(-5.438)	(-3.768)
*DUAL*	0.002	0.003*	0.022***	0.014***
	(1.192)	(1.716)	(5.841)	(4.486)
*Constant*	6.145***	6.116***	4.729***	5.277***
	(288.160)	(255.649)	(23.230)	(37.575)
*Year*	NO	YES	NO	YES
*Industry*	NO	YES	NO	YES
*Adj. R*^*2*^	0.1535	0.1735	0.1955	0.2148
*N*	26056	26056	8429	8429

***, **, and * indicate that the test statistics are significant at 1 %, 5 %, and 10 %, respectively.

### 5.2 The moderating effect of internal control willingness

The moderating effect of internal control willingness is reported in Table 4. In the analysis of the full sample, the coefficient of *F_POSITIVE* is insignificant, indicating that the moderating effect cannot be confirmed. This may be attributed to potential interference from non-foreign ownership samples, as foreign-owned firms only comprise 32.35 % of the sample. Thus, a substantial portion of non-foreign ownership samples resulted in more than 70 % of the *F_POSITIVE* variable having a value of 0. To mitigate this concern, a separate analysis is conducted on the subsample of 8429 foreign-owned firms, as illustrated in columns (3) and (4). The regression outcomes reveal that, regardless of industry and year effects, the coefficient of *F_POSITIVE* is significantly greater than 0. This affirms the moderating impact of internal control willingness, thereby validating hypothesis 2. In other words, in firms with positive internal control willingness, foreign investors actively contribute to shaping effective cooperation and jointly enhancing the efficiency and effectiveness of internal control mechanisms. Consequently, the beneficial influence of foreign ownership on internal control becomes more pronounced.

**Table 4:** The results of the moderating effect of internal control willingness.

Variable	Full sample		Sub-sample of Foreign Ownership	
	（1）	（2）	（3）	（4）
*F_POSITIVE*	−0.002	−0.003	0.051***	0.034*
	(-0.167)	(-0.227)	(2.604)	(1.764)
*FSHARE*	0.023***	0.021***	0.023**	0.017*
	(2.937)	(2.729)	(2.410)	(1.802)
*POSITIVE*	0.010***	0.002	−0.003	−0.008
	(5.073)	(1.020)	(-0.671)	(-1.611)
*SIZE*	0.014***	0.019***	0.020***	0.024***
	(15.349)	(20.032)	(13.916)	(16.671)
*LEV*	−0.030***	−0.039***	−0.023**	−0.038***
	(-5.162)	(-6.233)	(-2.157)	(-3.356)
*AGE*	−0.010***	−0.018***	−0.004	−0.015***
	(-2.875)	(-5.164)	(-0.771)	(-2.656)
*ROE*	0.388***	0.373***	0.420***	0.411***
	(36.598)	(35.071)	(20.419)	(19.919)
*PB*	0.001	0.000	0.001	0.002
	(0.904)	(0.671)	(1.018)	(1.452)
*TOBINQ*	−0.002	−0.000	0.001	0.002
	(-1.244)	(-0.230)	(0.458)	(0.816)
*TOP5*	0.055***	0.053***	0.051***	0.038***
	(9.281)	(8.668)	(4.934)	(3.542)
*BOARD*	0.002***	0.000	0.001	−0.000
	(2.938)	(0.205)	(1.161)	(-0.379)
*INDEP*	0.042**	0.024	0.011	−0.016
	(2.420)	(1.400)	(0.388)	(-0.547)
*BALANCE*	0.000	−0.000	−0.001**	−0.002***
	(1.109)	(-0.631)	(-2.402)	(-2.852)
*DUAL*	0.002	0.003*	0.006**	0.008***
	(1.075)	(1.694)	(2.078)	(2.754)
*Constant*	6.126***	6.113***	5.985***	6.015***
	(283.086)	(252.454)	(180.701)	(147.053)
*Year*	NO	YES	NO	YES
*Industry*	NO	YES	NO	YES
*Adj. R*^*2*^	0.1543	0.1735	0.1920	0.2127
*N*	26056	26056	8429	8429

***, **, and * indicate that the test statistics are significant at 1 %, 5 % and 10 %, respectively.

## 6 Robustness tests

### 6.1 Change in regression sample: propensity score matching method

Existing research has highlighted that foreign investors exhibit preferences in their investment choices [62,63], which may lead to sample selection bias in foreign investment firms. To mitigate the issue of sample selection bias, this study uses the propensity score matching method. Various variables potentially related to foreign investment decisions, such as firm size, leverage ratio, firm age, return on equity, price-to-book ratio, Tobin's Q ratio, the top five largest shareholders, board size, proportion of independent directors, ownership balance, and dual roles, are taken into consideration. Subsequently, we calculate propensity scores and apply a caliper of 0.05, ensuring a 1:1 ratio for matching. This process results in 7100 treatment group samples and 7100 control group samples. Balance test results indicate that the standardized deviations of each variable after matching are all within 10 %, signifying that there exists no vital difference between the matched groups, consistent with the balancing assumption of propensity score matching. The first column in Table 5 presents the regression results for the sample after propensity score matching. When simultaneously controlling for firm characteristics, industry, and year effects, the coefficient of *FSHARE* is 0.014, which is statistically significant at the 10 % level. The results confirm a positive correlation between foreign ownership and internal control in the revised regression sample.

**Table 5:** The results of the robustness test.

Variable	PSM	Tobit	Lag	ICA
	（1）	（2）	（3）	（4）
*FSHARE*	0.014*	0.020***		0.010*
	(1.823)	(2.610)		(1.661)
*FLAG*			0.014*	
			(1.843)	
*SIZE*	0.015***	0.019***	0.019***	0.000
	(10.330)	(20.486)	(19.952)	(0.154)
*LEV*	−0.015*	−0.039***	−0.024***	−0.026***
	(-1.700)	(-6.836)	(-3.651)	(-3.615)
*AGE*	−0.020***	−0.019***	−0.014***	−0.011***
	(-4.168)	(-5.214)	(-3.902)	(-2.636)
*ROE*	0.397***	0.373***	0.342***	0.107***
	(24.964)	(52.907)	(32.688)	(6.579)
*PB*	−0.000	0.001	0.000	−0.001*
	(-0.396)	(0.902)	(0.455)	(-1.766)
*TOBINQ*	0.002	−0.000	0.001	−0.001
	(0.982)	(-0.304)	(0.904)	(-0.628)
*TOP5*	0.045***	0.053***	0.040***	0.017**
	(5.248)	(8.845)	(6.687)	(2.485)
*BOARD*	−0.000	0.000	−0.000	0.001
	(-0.207)	(0.184)	(-0.357)	(0.872)
*INDEP*	−0.002	0.024	0.015	−0.012
	(-0.078)	(1.432)	(0.841)	(-0.583)
*BALANCE*	−0.000	−0.000	0.000	−0.000
	(-0.937)	(-0.737)	(0.164)	(-0.089)
*DUAL*	0.003	0.003*	0.000	0.001
	(1.297)	(1.688)	(0.142)	(0.506)
*Constant*	6.206***	6.116***	6.073***	1.023***
	(166.374)	(265.127)	(248.816)	(41.118)
*Year*	YES	YES	YES	YES
*Industry*	YES	YES	YES	YES
*Adj. R*^*2*^*/Pseudo R*^*2*^	0.1532	−0.1754	0.1729	0.0188
*N*	14200	26056	21244	19875

***, **, and * indicate that the test statistics are significant at 1 %, 5 % and 10 %, respectively.

### 6.2 Change in regression models: Tobit model

Since the dependent variable, the internal control level, is non-negative, potential censoring issues may arise. To address this concern, we employed the Tobit model for analysis. The robustness test results, presented in columns (2) of Table 5, reflect the effects of changing the regression model. The coefficient derived from the Tobit model demonstrates a significant positive association between *FSHARE* and *IC* at the 1 % confidence level. Therefore, the empirical results from the alternative regression models further bolster the findings obtained from the baseline regression analysis.

### 6.3 Change in explanatory variable: lagged regression

Due to the investment preferences of foreign shareholders, endogeneity issues might arise. To solve this problem, we replace the explanatory variable with the lagged foreign shareholding percentage (*FLAG*) variable. The results of the lagged regression are presented in column (3). Controlling for firm characteristics, industry, and year effects, the coefficient of *FLAG* is found to be significantly positive. This finding contributes to alleviating endogeneity concerns to some extent.

### 6.4 Change in dependent variable: internal control audit opinion

This section introduces the Internal Control Audit Opinion (*ICA*) as a replacement for the dependent variable, the Internal Control Level. When the internal control audit opinion is a standard unqualified opinion, we assign a value of 1 to *ICA*. If the opinion type differs from unqualified, such as unqualified opinions with emphasis on matter paragraphs, disclaimers of opinion, or adverse opinions, we assign a value of 0. According to the result presented in column (4), foreign ownership is positively correlated with the Internal Control Audit Opinion, which is significant at a 10 % level. These results align with the fundamental research conclusions.

## 7 Further analysis

### 7.1 Further effects on sustainable development

Sustainable development, introduced in the 1987 report “Our Common Future”, involves balancing economic, social, resource, and environmental concerns, which gained widespread global recognition [64]. Increasingly, Environmental, Social, and Governance (ESG) principles have become the core concept of sustainable development. Strong ESG performance contributes to reducing environmental impact [65], driving governance reforms, creating corporate value, and promoting societal sustainability [66]. These factors collectively promote the long-term sustainability and success of firms.

Prior research suggests that the positive impact of foreign shareholders extends beyond internal control quality, profoundly shaping the overall trajectory of host country companies, particularly in sustainable development [10,67]. Foreign shareholders actively drive an ‘inside-out’ transformation, commencing with improvements in internal control mechanisms and culminating in the enhanced operational environment and competitive advantage [68]. This improvement not only strengthens the capacity for sustainable development but also fosters positive synergy among businesses, the environment, and society, establishing a solid foundation for future sustainability. That is to say, foreign shareholders, by improving internal control, advance responsible and sustainable business practices, aligning with ESG principles [48,69]. Therefore, our study will explore the further effect of foreign ownership on sustainable development.

Using the stepwise regression method based on the mediating effect, this study assesses the impact of foreign shareholders on sustainable development by considering three factors within the Huazheng ESG evaluation data. The baseline regression results are reported in column (1), while columns (2)–(3), (4)–(5), and (6)–(7) present the regression results related to environmental, social, and governance factors, as presented in Table 6.

**Table 6:** The results of further effects on sustainable development.

Variable	IC	Environmental		Social		Governance	
	（1）	（2）	（3）	（4）	（5）	（6）	（7）
*FSHARE*	0.021***	0.259***	0.253***	0.076	0.047	0.296***	0.250***
	(3.007)	(3.699)	(3.615)	(1.210)	(0.752)	(4.117)	(3.524)
*IC*			0.292***		1.427***		2.252***
			(5.218)		(22.865)		(26.667)
*SIZE*	0.018***	0.197***	0.192***	0.238***	0.211***	0.203***	0.161***
	(19.874)	(22.966)	(22.197)	(31.849)	(28.259)	(22.812)	(18.298)
*LEV*	−0.036***	0.383***	0.394***	−1.006***	−0.954***	−2.247***	−2.165***
	(-5.834)	(7.669)	(7.876)	(-21.605)	(-20.819)	(-38.881)	(-38.496)
*AGE*	−0.018***	0.043	0.048	−0.127***	−0.100***	0.028	0.069**
	(-5.196)	(1.389)	(1.563)	(-4.303)	(-3.454)	(0.785)	(2.011)
*ROE*	0.369***	0.271***	0.163**	1.269***	0.743***	1.354***	0.523***
	(34.591)	(4.142)	(2.405)	(19.186)	(11.005)	(15.981)	(6.096)
*PB*	0.000	−0.029***	−0.029***	−0.003	−0.004	0.015**	0.014**
	(0.582)	(-6.482)	(-6.526)	(-0.603)	(-0.757)	(2.300)	(2.285)
*TOBINQ*	0.001	−0.004	−0.004	−0.032***	−0.033***	−0.020	−0.021*
	(0.427)	(-0.430)	(-0.451)	(-3.136)	(-3.322)	(-1.518)	(-1.710)
*TOP5*	0.051***	−0.087	−0.102*	0.355***	0.282***	0.692***	0.577***
	(8.398)	(-1.638)	(-1.915)	(7.272)	(5.872)	(11.577)	(9.929)
*BOARD*	−0.000	−0.005	−0.005	0.026***	0.026***	0.041***	0.041***
	(-0.007)	(-0.917)	(-0.918)	(5.217)	(5.333)	(7.132)	(7.321)
*INDEP*	0.024	−0.152	−0.159	1.854***	1.820***	3.875***	3.822***
	(1.357)	(-1.007)	(-1.053)	(13.435)	(13.453)	(23.671)	(23.901)
*BALANCE*	−0.000	−0.001	−0.000	−0.004**	−0.004*	0.013***	0.014***
	(-0.792)	(-0.262)	(-0.233)	(-1.986)	(-1.863)	(5.641)	(5.986)
*DUAL*	0.003*	−0.066***	−0.067***	−0.050***	−0.055***	−0.083***	−0.090***
	(1.880)	(-4.015)	(-4.077)	(-3.364)	(-3.748)	(-4.470)	(-5.020)
*Constant*	6.119***	−1.986***	−3.773***	−1.613***	−10.348***	0.848***	−12.930***
	(254.071)	(-9.517)	(-9.435)	(-8.644)	(-24.504)	(3.491)	(-22.629)
*Year*	YES	YES	YES	YES	YES	YES	YES
*Industry*	YES	YES	YES	YES	YES	YES	YES
*Adj.R*^*2*^	0.1707	0.1134	0.1143	0.1544	0.1806	0.2060	0.2469
*N*	25601	25601	25601	25601	25601	25601	25601

***, **, and * indicate that the test statistics are significant at 1 %, 5 % and 10 %, respectively.

Columns (1)–(3) highlight a significant enhancement in the impact of foreign ownership on environmental development attributed to improvements in the internal control system. Additionally, columns (1), (6), and (7) reveal that the promotion of corporate governance mechanisms occurs through the improvement of internal control quality. However, regarding social aspects, the presence of a partial mediating effect cannot be firmly established, as shown in columns (1), (4) and (5). One possible explanation is that social development encompasses a wide array of stakeholders and complex factors, such as cultural, ideological, and political elements. While previous research has highlighted the positive influence of corporate social responsibility [70], their ability to profoundly impact other facets of social development may be constrained. Consequently, while foreign shareholders can influence corporate social responsibility to some extent, their broader impact on societal development may be limited, resulting in the absence of a mediating effect.

### 7.2 Moderating effect of foreign shareholder types

Foreign investors can be typically categorized into legal entities and individuals. Generally, individuals tend to have less professional expertise and are less involved in decision-making compared to legal entity shareholders [43]. As a result, their level of participation in governance activities, such as internal control, may be lower. In contrast, legal entity shareholders are usually more actively engaged in corporate governance [71,72]. They generally have greater financial resources and higher ownership stakes, affording them a certain degree of influence over the development of internal control. Additionally, given that internal control systems in foreign countries have evolved earlier than in China, legal entity shareholders are likely to bring valuable experiences. This, in turn, is beneficial for enhancing the internal control standards of Chinese firms.

To explore this further, this study introduces two dummy variables: *INDIV* and *CORPOR*. The *INDIV* variable is assigned a value of 1 when individual foreign shareholders are among the top ten shareholders; otherwise, it is assigned 0. Similarly, the *CORPOR* variable is created. Furthermore, the heterogeneity impact of foreign shareholder types is examined based on Models (3)–(5), with the regression results presented in Table 7.

(3) $$\begin{array}{c} {IC}_{i,t}=\alpha_{0}+\alpha_{1}{\;INDIV}_{i,t}+\alpha_{2\;}{Control}_{i,t}+\sum INDUSTRY+\sum YEAR+\varepsilon_{i,t} \end{array}$$

(4) $$\begin{array}{c} {IC}_{i,t}=\alpha_{0}+\alpha_{1}{\;CORPOR}_{i,t}+\alpha_{2\;}{Control}_{i,t}+\sum INDUSTRY+\sum YEAR+\varepsilon_{i,t} \end{array}$$

(5) $$\begin{array}{c} {IC}_{i,t}=\alpha_{0}+\alpha_{1}{\;INDIV}_{i,t}+\alpha_{2}{\;CORPOR}_{i,t}+\alpha_{3\;}{Control}_{i,t}+\sum INDUSTRY+\sum YEAR+\varepsilon_{i,t} \end{array}$$

**Table 7:** The results of moderating effect of foreign shareholder types.

Variable	（1）	（2）	（3）
*INDIV*	−0.000		−0.001
	(-0.051)		(-0.288)
*CORPOR*		0.005***	0.005***
		(2.754)	(2.768)
*SIZE*	0.019***	0.018***	0.018***
	(20.483)	(18.479)	(18.477)
*LEV*	−0.039***	−0.038***	−0.038***
	(-6.336)	(-6.095)	(-6.095)
*AGE*	−0.019***	−0.019***	−0.019***
	(-5.255)	(-5.333)	(-5.332)
*ROE*	0.373***	0.372***	0.372***
	(35.049)	(34.938)	(34.929)
*PB*	0.000	0.000	0.000
	(0.673)	(0.614)	(0.617)
*TOBINQ*	−0.000	−0.000	−0.000
	(-0.135)	(-0.317)	(-0.321)
*TOP5*	0.057***	0.055***	0.055***
	(9.614)	(9.193)	(9.198)
*BOARD*	0.000	0.000	0.000
	(0.196)	(0.181)	(0.177)
*INDEP*	0.024	0.025	0.025
	(1.407)	(1.420)	(1.418)
*BALANCE*	−0.000	−0.000	−0.000
	(-0.873)	(-0.813)	(-0.813)
*DUAL*	0.003*	0.003*	0.003*
	(1.770)	(1.690)	(1.692)
*Constant*	6.109***	6.128***	6.129***
	(257.784)	(245.929)	(245.976)
*Year*	YES	YES	YES
*Industry*	YES	YES	YES
*Adj.R*^*2*^	0.1733	0.1736	0.1736
*N*	26056	26056	26056

***, **, and * indicate that the test statistics are significant at 1 %, 5 % and 10 %, respectively.

The coefficient in column (1) confirms our earlier analysis, showing that individual foreign shareholders exhibit lower attention and weaker influence on the host country firm, resulting in an insignificant impact on the internal control level. Conversely, in column (2), the coefficient of *CORPOR* is positive at a 1 % confidence level, suggesting that legal entity foreign shareholders are more willing to engage in the firm's operational decision-making, thereby encouraging management to bolster the internal control framework. Column (3) presents the combined results of testing both dummy variables simultaneously, reinforcing the conclusions drawn from the individual analyses. Consequently, it becomes evident that the entry of legal entity foreign shareholders into a host country firm significantly enhances the internal control levels.

### 7.3 Moderating effect of industry competition level

Competition serves four key functions: motivating effort, improving information, prompting development, and evolving markets [73]. Within the sphere of internal control, market competition plays a pivotal role and can lead to differences in various aspects of internal control practices [74]. In industries characterized by intense competition, firms encounter challenges in gaining a competitive edge due to the prevalence of numerous homogeneous competitors [75,76]. Faced with survival challenges, shareholders focus excessively on financial performance, compelling professional managers to improve operational efficiency to maximize shareholder interests continually. In this context, an effective way to cope with the pressure and achieve sustained growth is to enhance internal control quality.

On the contrary, in a less competitive environment marked by fewer companies or greater market differentiation, the comparability of financial information diminishes, and operational uncertainties decrease. Due to cost-benefit considerations, management may be content with the current market share and reluctance to alter the existing internal control system. As a result, the positive effect of foreign ownership on internal control may be less pronounced in firms operating in low-competition industries.

Industry competition is quantified using the Herfindahl-Hirschman Index (*HHI*), with a smaller *HHI* denoting more intense competition. Samples are classified based on the median *HHI*, with those falling below the median categorized as high-competition and those surpassing it deemed low-competition. The results in columns (1) and (2) of Table 8 demonstrate that only in the high-competition industries is the coefficient of *FSHARE* significantly positive. It suggests that the impact of foreign ownership on improving internal control is contingent upon industry competition levels. Intense competition may motivate foreign shareholders to leverage their advantage, significantly enhancing the internal control level of host country firms.

**Table 8:** The results of moderating effects of industry competition level and regional legal environment.

Variable	Industry Competition Level		Regional Legal Environment	
	High	Low	Favorable	Weak
	（1）	（2）	（3）	（4）
*FSHARE*	0.027***	0.011	0.019**	0.012
	(2.987)	(1.126)	(2.400)	(1.006)
*SIZE*	0.015***	0.022***	0.017***	0.019***
	(10.810)	(16.843)	(14.448)	(13.042)
*LEV*	−0.038***	−0.043***	−0.006	−0.059***
	(-4.432)	(-4.642)	(-0.703)	(-6.721)
*AGE*	−0.011**	−0.027***	−0.013***	−0.024***
	(-2.117)	(-5.464)	(-2.753)	(-4.341)
*ROE*	0.400***	0.350***	0.361***	0.387***
	(25.924)	(23.920)	(24.607)	(25.124)
*PB*	0.000	0.001	0.001	−0.000
	(0.014)	(1.030)	(0.894)	(-0.339)
*TOBINQ*	−0.001	0.001	0.000	−0.000
	(-0.441)	(0.371)	(0.240)	(-0.237)
*TOP5*	0.051***	0.049***	0.071***	0.028***
	(5.977)	(5.546)	(8.263)	(3.228)
*BOARD*	0.001	−0.001	0.001	−0.000
	(1.354)	(-0.882)	(0.793)	(-0.183)
*INDEP*	0.014	0.030	0.028	0.019
	(0.530)	(1.268)	(1.166)	(0.782)
*BALANCE*	−0.000	−0.000	0.000	−0.000
	(-0.341)	(-0.803)	(0.452)	(-0.830)
*DUAL*	0.001	0.005*	0.005*	0.000
	(0.606)	(1.835)	(1.903)	(0.024)
*Constant*	6.199***	6.062***	6.115***	6.141***
	(181.787)	(190.509)	(155.818)	(173.210)
*Year*	YES	YES	YES	YES
*Industry*	YES	YES	YES	YES
*Adj.R*^*2*^	0.1853	0.1690	0.1770	0.1788
*N*	13098	12955	12977	13076

***, **, and * indicate that the test statistics are significant at 1 %, 5 % and 10 %, respectively.

### 7.4 Moderating effect of regional legal environment

The market environment encompasses the external institutional conditions that firms encounter, with its degree playing a vital role in determining the effectiveness of institutional investors' governance practices [77]. The regional legal environment, as a key indicator of marketization, acts as a moderating role in shaping the behavior of foreign shareholders. Sound legal systems and regulatory conditions impose mandatory constraints on information disclosure and contribute, thus safeguarding investors’ interests [78]. In regions with a favorable legal environment, the information environment is transparent, and managers demonstrate strong compliance with internal control standards. Consequently, investors can more accurately identify internal control deficiencies and provide constructive suggestions efficiently.

Conversely, regions with weaker legal systems often witness higher incidences of accounting fraud and earnings manipulation. In such environments, foreign shareholders encounter severe challenges in identifying internal control deficiencies. Moreover, management and controlling shareholders may impede foreign shareholders’ involvement in corporate governance. Even if the manager is committed to improving the internal control level, resource constraints may restrict their ability to address agency problems and enhance internal control quality simultaneously.

To examine the impact of the regional legal environment, we utilize the “Development of Market Intermediaries and Legal System Score” to gauge the regional legal environment (*LAW*). Based on the median scores, this study categorizes regions into two groups: those with a favorable legal environment and those with a less favorable legal environment. Regression results are performed on these sub-samples, with the results detailed in columns (3) and (4) of Table 8. In the sub-sample with a favorable legal environment, there is a positive relationship between foreign ownership and internal control. However, in regions with weaker legal environments, the coefficient of *FSHARE* is not statistically significant. These empirical results confirm that, compared to regions with weaker legal systems, the positive impact is more pronounced in regions with a more robust legal environment.

## 8 Conclusions, implications, limitations, and further research

### 8.1 Conclusions

In the midst of China's vigorous efforts to attract foreign investment, understanding the behavioral characteristics and economic effects of foreign ownership holds major significance for the country's economic development. Our study integrates foreign ownership and internal control quality into a unified analytical framework, revealing the following key findings. First, foreign ownership significantly enhances the internal control levels of host country firms. Second, the willingness for internal control moderates the positive effect of foreign shareholding. In firms with a positive willingness, foreign investors prove more adept at enhancing internal control quality. Furthermore, detailed analysis suggests that the impact on internal control ultimately contributes to the advancement of firms' sustainable development. Additionally, when foreign shareholders are legal entities operating in highly competitive industries or a favorable regional legal environment, foreign investors' impact is more pronounced.

### 8.2 Implications

The conclusions drawn from this study enrich the theoretical research on the relationship between foreign ownership and internal control, providing substantial implications for shaping and optimizing national policies related to foreign investment. In an era of economic globalization, the gradual opening to foreign investment is undeniable. Active participation of foreign shareholders in corporate governance positively impacts controlling opportunistic behavior and improving internal control quality within listed companies. These actions contribute to boosting the overall integrity and transparency of China's capital market, which can, in turn, attract more foreign investment and promote sustainable economic growth. The relationship between foreign ownership, internal control, and sustainable development is critical, requiring ongoing research and attention as China's economy and global integration evolve. Based on these findings, the following recommendations are proposed:

First, facilitate channels for foreign investment. The government might consider simplifying procedures and reducing barriers to investment, thereby attracting more overseas capital into our domestic market. Additionally, our country could also actively create a favorable and stable business environment, promote fair and orderly market competition, and thus enhance its attractiveness to overseas investors.

Second, safeguard the legitimate rights and interests of stakeholders. The policymakers might establish a sound investor protection system, ensuring necessary legal safeguards for overseas investors and preventing violations of their legitimate rights and interests. Simultaneously, relevant authorities should enhance regulations in the field of foreign shareholding, strengthen supervision of foreign investment, and remain vigilant against shortfall, thereby promoting stable and positive development in various aspects of local companies.

Third, guide foreign shareholders to exert positive effects. The government should encourage foreign shareholders to actively participate in internal governance, foster communication and information exchange with domestic firms, promote international cooperation, and strive to improve internal control quality. The country can also provide professional consultancy or training to overseas investors, helping foreign shareholders better understand and integrate into the internal governance of domestic firms, jointly promoting sustainable development.

In the end, domestic firms should fully recognize the importance of foreign investment, actively expressing an open attitude towards accepting overseas investment. Upon introducing overseas investors, local companies can benefit from the advanced resources and technologies, proactively integrating them to gain sustainable competitive advantages.

### 8.3 Limitations and further research

The internal control willingness (*POSITIVE*) variable holds higher credibility for firms that publicly disclose their internal control self-assessment reports. However, it is worth noting that some firms have chosen not to disclose their self-assessment reports, which poses certain limitations in the sample when measuring. Therefore, it is advisable to consider conducting sentiment analysis on internal control willingness based on mandatory disclosure reports in future research. This approach can help address the missing data in the sample and provide valuable insights into the internal control willingness of those who have not disclosed their self-assessment reports.

Additionally, there is a need for further research on the impact of foreign ownership on social development. While our study has provided theoretical support for this assertion, it is important to recognize that social responsibility encompasses many factors, and foreign ownership's influence on enhancing social responsibility may necessitate an extended observation period. However, these theoretical explanations have not yet been empirically validated on a large-scale sample. Future researchers can delve deeper into this direction to examine the relationship between foreign ownership and various aspects of social responsibility. It will contribute to the enrichment of research in the field of sustainability and provide a more robust understanding of how foreign shareholders influence corporate social responsibility practices.

## Data availability statement

The data supporting this study's findings are available from the corresponding author upon reasonable request.

## Funding information

The author(s) disclosed receipt of the following financial support for the research, authorship, and/or publication of this manuscript:

Study on the Course Module Design and Teaching Model Reform for Training Excellent Accounting Talents under the Convergence of ‘Dual Carbon’ Goals and ‘Digital Economy’—a Graduate Teaching Reform Project of the Zhejiang Province's 14th Five-Year Plan.

General Research Project of 10.13039/501100008867Zhejiang Provincial Department of Education (No. 2023-005) and the Zhejiang Graduate Education Society (No. 2023-005).

## CRediT authorship contribution statement

**Yanxin Yi:** Supervision. **Qin Yao:** Writing – original draft. **Wunhong Su:** Writing – review & editing.

## Declaration of competing interest

All authors declare that No conflict of interest exists.

## Footnotes

1 The disclosure of internal control self-assessment reports for 2012 to 2021 occurred between 2013 and 2022.

2 This study applies the TSNE technique to visualize multi-dimensional features clustered into three-dimensional space for further analysis.
